# Artificial Intelligence in Food Safety: A Tertiary Study

**DOI:** 10.1111/1541-4337.70443

**Published:** 2026-03-14

**Authors:** Marina Arribas Lopez, Yamine Bouzembrak, Bedir Tekinerdogan

**Affiliations:** ^1^ Information Technology Group Wageningen University & Research Wageningen the Netherlands

## Abstract

Food safety remains a critical factor in preventing contaminated and hazardous products from reaching consumers. The integration of artificial intelligence (AI) and its capacity to deal with vast datasets has significantly enhanced food safety protocols, and a substantial number of primary and secondary studies have emerged at the intersection of these two domains. Although several studies have addressed AI applications in food safety, no tertiary study has yet synthesized the collective insights from existing systematic reviews. To address this gap, this paper provides a comprehensive overview of the current state of AI applications in food safety through a systematic tertiary analysis of secondary studies. By systematically analyzing secondary studies, this research identifies key trends such as the food categories most frequently investigated, the data sources utilized, prevalent food safety hazards, the commonly adopted AI algorithms, and the challenges associated with their implementation within the field. The analysis revealed that dairy products received the greatest research attention, with sensing data serving as the primary data source. Neural networks emerged as the predominant AI approach. Furthermore, most applications focused on the detection of chemical food safety hazards rather than biological, physical, or general predictive modeling. Notably, this study highlights a lack of AI algorithms utilizing unstructured data, despite its growing relevance in the era of generative AI. Accordingly, future research directions are discussed, particularly the transformative potential of large language models (LLMs) in food safety monitoring and regulatory compliance.

## 1 Introduction

Ensuring access to safe and nutritious food is essential for sustaining life and promoting optimal public health. The consumption of contaminated products results in more than 200 diseases, including foodborne illnesses that can lead to long‐lasting disability and mortality. Approximately one in three consumers contracts a foodborne disease associated with microbes or their toxins every year in developed countries; furthermore, as the food supply chain becomes more globalized, the potential for massive foodborne outbreaks intensifies, as do the difficulties in mitigating their widespread effects (Bricher 2022). Therefore, maintaining rigorous food safety (FS) protocols is paramount for human health and survival, needing the implementation of more advanced monitoring and control mechanisms.

FS encompasses all the processes or actions that prevent food from containing substances that could harm a person's health (FAO 2023). These substances, referred to as FS hazards are typically classified into three different categories: (i) biological hazards, comprising bacteria, parasites, fungi, and viruses; (ii) chemical hazards, which include toxins, either intrinsic to food or secreted by microorganisms, such as mycotoxins or biogenic amines, as well as anthropogenic contaminants such as pesticides, veterinary drugs, environmental pollutants, and heavy metals, in addition yo compounds generated during food procesing; and (iii) physical hazards, including metal fragments and glass debris (FDA 2018). To detect these potential contaminants, several advanced techniques, including biosensors, hyperspectral imaging (HSI), and ultraviolet (UV) photography, are employed within FS monitoring plans across all stages of the supply chain (Aggarwal et al. 2024). However, the resulting data volumes require the use of high‐throughput computational methods, like artificial intelligence (AI), to extract meaningful insights essential for the prevention or prediction of FS issues (Z. Liu et al. 2023; Mu et al. 2024).

In recent years, scholarly attention has shifted increasingly toward AI technologies, leading to significant advancements across all sectors of the food industry. AI algorithms possess the capability to derive insight, knowledge, and patterns from heterogeneous data, ranging from numerical metrics to unstructured textual information, to generate results ranging from simple data grouping to more advanced outcomes, such as statistical probabilities, classifications, or predictive modeling (Tajkarimi 2020). In addition, these technologies are adept at processing high‐dimensional datasets and a diverse set of variables, rendering them suitable for a wide variety of purposes. Key applications include enhancing food production, quality, and nutrition, more efficient supply chain management, and reducing resource consumption and waste (Z. Liu et al. 2023).

Consequently, AI is poised to play a pivotal role in improving FS by offering solutions for the proactive detection of FS hazards, facilitating efficient risk assessment, and enhancing regulatory compliance throughout the food industry. Furthermore, AI can generate actionable insights specifically for high‐risk groups, enabling specialized guidance for vulnerable subpopulations such as those at risk due to genetic predispositions or disease.

The volume of scientific literature pertaining to the intersection of FS and AI is extensive; consequently, the number of secondary studies conducted on this topic is considerable. However, to manage the vast scope of these two research areas, existing reviews frequently limit their focus to narrow sub‐domains. These range from analysis of specific techniques such as HSI in FS and how AI optimizes its performance (Gul et al. 2024), to the application of AI within specific supply chains, such as fruits and vegetables (Yuvaraj et al. 2023). This fragmentation limits the ability to develop an integrated understanding of AI's overarching contribution to FS. Therefore, a tertiary study is imperative to synthesize and consolidate the insights from these diverse secondary studies, evaluate their methodological rigor, and identify remaining research gaps. Unlike a secondary study, which analyzes individual primary research, a tertiary study provides a meta‐level perspective on the systematic rigor and comprehensiveness with which AI applications in FS have been reviewed to date. This establishes a clear baseline for future research and guides the development of more focused, methodologically consistent reviews. The need for a tertiary study is further supported by the current literature. For example, Z. Liu et al. (2023) emphasized that despite the increasing integration of AI across various fields within FS, significant gaps remain in both the depth and breadth of its applications. Similarly, Raki et al. (2024) highlighted that FS intersects multiple domains of research and industry; although their study primarily focused on crop‐based foods, they stressed that food contamination prevention remains a persistent challenge with multiple hazards involved.

A tertiary study systematically synthesizes data from existing secondary studies, offering a holistic review of the research undertaken within a specific domain (Ligthart et al. 2021). Although tertiary studies have been conducted across various disciplines, such as sentiment analysis (Ligthart et al. 2021) and software engineering (Cadavid et al. 2020), as well as in the agri‐food sector, notably in precision livestock farming (Distante et al. 2025) and supply chains (Barata 2021), to date, no tertiary study has integrated then fields of FS and AI.

Consequently, the objective of this study is to identify and characterize extant secondary studies focusing on the application of AI techniques to enhance FS. The methodology employed adhered to the guidelines proposed by Kitchenham et al. (2009), which align closely with the protocols for systematic literature reviews (SLRs). This systematic approach is crucial for identifying perspective areas for future research in this domain (Ligthart et al. 2021).

The main contributions of this article are threefold: (i) we present the findings of the inaugural tertiary study within the literature on AI applications in FS; (ii) we identify extant reviews and select those that comply with rigorous methodological standards; and (iii) we provide systematic insights into the most frequently investigated food products, the predominant AI algorithms, and the most commonly addressed FS hazards.

This article is organized as follows: Section 2 provides the theoretical background, Section 3 describes the research methodology, Section 4 presents the analytical results, Section 5 discusses the findings and outlines suggestions for future work, and Section 6 concludes the study.

## 2 Background

FS and AI are two expansive and rapidly evolving research fields situated within the domains of food science and computer science, respectively. This section provides a concise synthesis of these two concepts for readers who may lack familiarity with the domains. Given the inherent heterogeneity and scope of both areas, the following discussion is intended as an introductory overview rather than an exhaustive analysis.

### 2.1 Food Safety, Food Fraud, Food Defense, and Food Quality

According to the WHO, FS hazards are defined as biological, chemical, or physical agents in, or conditions of, food that can potentially cause an adverse health effect. The present study specifically focuses on FS by addressing the three primary categories of these hazards (i.e., biological, chemical, and physical).

A recent comprehensive review of food fraud (FF) terminology and mitigation guidelines (Robson et al. 2021) identified several distinct definitions of FF in the scientific literature. Despite some variation, most definitions converge on the idea that FF involves the intentional deception of consumers or stakeholders for economic gain using food products. One widely cited definition is provided by Spink et al. (2019), who describe FF as “Illegal deception for economic gain using food, encompassing deliberate substitution, addition, tampering, or misrepresentation of food, ingredients, or packaging, as well as false or misleading statements about a product for economic gain.” This definition also recognizes several types of fraud, including adulteration, tampering, product overrun, theft, diversion, simulation, and counterfeiting.

**FIGURE 1:**
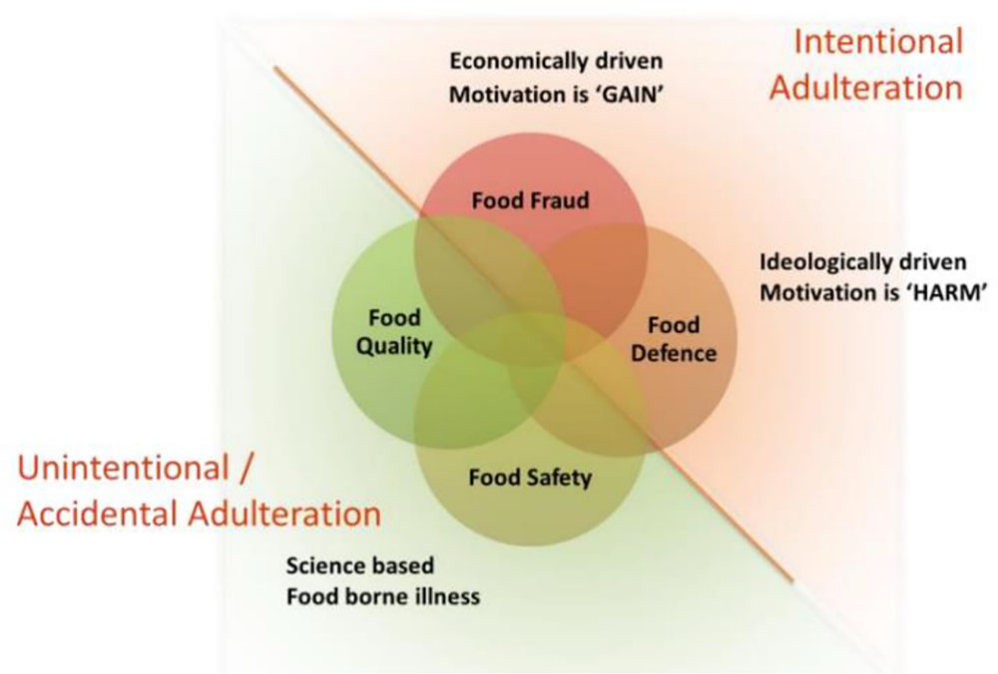
Unintentional and intentional adulterations in food. *Source*: FSSC 22000 (2023).

Within the context of food control systems, FF is characterized by nuances in motivation and intentionality, enabling a distinction between four primary elements. First, if the adulteration is deliberate and motivated by economic gain, it is classified as FF; for example, producers may mislabel the geographic origin of products to command premium pricing. Second, if the objective is to deliberately inflict harm (i.e., economic or health), the incident falls under food defense (FD), exemplified by the intentional incorporation of toxic substances into a product. Furthermore, FD also encompasses incidents designed to undermine consumer confidence in the capacity of FS authorities to provide adequate protection. Third, when adulteration arises unintentionally or accidentally and only diminishes sensory attributes such as color, taste, or texture, it falls under food quality (FQ). Conversely, accidental contamination that poses a direct risk to human health, potentially causing foodborne illness, constitutes the domain of FS (Manning and Soon 2016). An overview of these relationships is depicted in Figure 1.

### 2.2 Artificial Intelligence

AI has been extensively defined by researchers over the recent decades; its nature as an evolving concept necessitates continuous refinement of these descriptions. In 2024, the European Commission published an updated definition of AI, stating that an “Artificial intelligence (AI) system means a machine‐based system that is designed to operate with varying levels of autonomy and that may exhibit adaptiveness after deployment, and that, for explicit or implicit objectives, infers, form the input it receives, how to generate outputs such as predictions, content, recommendations, or decisions that can influence physical or virtual environments” (European Union 2024).

In summary, AI serves as an umbrella term encompassing a variety of algorithm‐based technologies designed to perform complex tasks that traditionally required human cognition (Distante et al. 2025). This study includes AI sub‐fields related to learning, communication, and perception, specifically machine learning (ML), natural language processing (NLP), and computer vision (CV), while maintaining a broad and holistic perspective. ML focuses on the development and implementation of algorithms designed to discern patterns from datasets with the goal of improving system performance through experience (Alpaydin 2020). It comprises various paradigms, the most prevalent being supervised learning, unsupervised learning, and reinforcement learning.

Deep learning (DL) involves more advanced ML methods, characterized by neural networks (NNs) with multiple layers that can extract higher‐level features (Friedlander and Zoellner 2020). DL is frequently utilized for CV applications, among which convolutional neural networks (CNNs) are among the most prevalent. Furthermore, DL is essential for NLP, which commonly relies on transformer architecture. Detailed information on the specific algorithms examined in this tertiary study is provided in Section 4.

## 3 Research Methodology

This section outlines the methodology employed for the tertiary study, which adheres to the principles of an SLR. As previously mentioned, the study design follows the guidelines proposed by Kitchenham et al. (2009). This framework encompasses a structured process comprising: (i) the identification of research questions; (ii) the formulation of search queries and database selection; (iii) the definition of inclusion and exclusion criteria; (iv) the assessment of article quality using standardized evaluation protocols; and finally, (v) data extraction from the studies that met all eligibility requirements. The methodology also aligns with the methodological format used in other tertiary studies, such as Ligthart et al. (2021).

### 3.1 Research Questions

The study is designed to address the following research questions. The first category (RQ1) adopts a meta‐level perspective, examining methodological aspects such as the article selection process, eligibility criteria, and the thematic scope of the extant research. The second category (RQ2) explores the thematic insights and findings derived from the analyzed secondary studies.

RQ 1.1 *What research methodologies are employed in the reviews analyzed in this study?*

RQ 1.2 *What is the scope of the reviews? Do they address general FS issues or focus on specific FS hazards, foods, or techniques?*

RQ 2.1 *To which specific foods or products has AI been most frequently applied in FS?*

RQ 2.2 *What types of data have been utilized alongside AI in the field of FS?*

RQ 2.3 *Which specific FS hazards have been addressed using AI?*

RQ 2.4 *Which AI algorithms or models are commonly used in FS‐related problems?*

RQ 2.5 *What are the key challenges regarding FS and AI?*

### 3.2 Search Process

The search process was strategically designed to identify extant secondary studies relevant to the integration of AI techniques within the domain of FS. The search query was constructed using a tripartite structure: (1) terms aimed at filtering results based on the primary thematic area (FS), (2) keywords targeting the specific application or techniques under investigation (AI, ML, or DL), and (3) descriptors intended to isolate document types categorized as SLRs or systematic mapping studies.

To ensure relevance and the contemporaneity of the findings, temporal parameters were established to include only studies published from 2020 to the present. The following bibliographic databases were consulted: PubMed, Scopus, Web of Science, IEEE Xplore, and ProQuest. The search query was executed across the title, abstract, and keywords as follows: (“food safety”) AND (“machine learning” OR “artificial intelligence” OR “AI” OR “deep learning”) AND (“review” OR “SLR” OR “systematic literature review” OR “systematic mapping” OR “mapping study”) AND (Publication Date: (January 1, 2020 TO December 31, 2024)).

Table 1 presents the initial hits retrieved by applying these search terms across each database, yielding a total of 764 candidate secondary studies. These records were subsequently subjected to the succeeding step of our selection process.

**TABLE 1:** The number of papers returned from each database with the search query.

Database	Search query	Hits
PubMed	(”food safety” [Title/Abstract]) AND (“machine learning” [Title/Abstract] OR “artificial intelligence” [Title/Abstract] OR “AI” [Title/Abstract] OR “deep learning” [Title/Abstract]) AND (“review” [Title/Abstract] OR “SLR” [Title/Abstract] OR “systematic literature review” [Title/Abstract] OR “systematic mapping” [Title/Abstract] OR “mapping study” [Title/Abstract])	60
Scopus	TITLE‐ABS‐KEY ((“food safety”) AND (“machine learning” OR “artificial intelligence” OR “AI” OR “deep learning”) AND (“review” OR “SLR” OR “systematic literature review” OR “systematic mapping” OR “mapping study”)) AND PUBYEAR ¿ 2019 AND PUBYEAR ¡ 2026	278
Web of Science	TS = (“food safety” AND (“machine learning” OR “artificial intelligence” OR “AI” OR “deep learning”) AND (“review” OR “SLR” OR “systematic literature review” OR “systematic mapping” OR “mapping study”)) AND PY = (2020–2025)	162
IEEE	(“All Metadata”: food safety) AND (“All Metadata”: machine learning OR “All Metadata”: AI OR “All Metadata”: artificial intelligence OR “All Metadata”: deep learning) AND (“All Metadata”: review OR “All Metadata”: SLR OR “All Metadata”: systematic literature review OR “All Metadata”: systematic mapping OR “All Metadata”: mapping study)	69
ProQuest	ABSTRACT, TITLE (“food safety”) AND ABSTRACT, TI‐ TLE (“machine learning” OR “artificial intelligence” OR “AI” OR “deep learning”) AND ABSTRACT, TITLE (“review” OR “SLR” OR “systematic literature review” OR “systematic mapping” OR “mapping study”)	155
Total		724

**TABLE 2:** Inclusion and exclusion criteria.

Inclusion criteria	
IC1: The paper is a literature review	
IC2: The review is related to FS and includes/mentions the use of AI models	
Exclusion criteria	
EC1: The paper is not considered a secondary study. This excludes conferences, books, book chapters	
EC2: The secondary study is not directly related to FS, out of scope	
EC3: The paper does not include examples of algorithms	
EC4: The paper is not written in English	
EC5: The study is a duplicate of another paper in the sample	

### 3.3 Inclusion and Exclusion Criteria

The subsequent phase involves the formulation of objective inclusion and exclusion criteria, as detailed in Table 2. These criteria were applied systematically to the 764 candidate secondary studies ensure the reproducibility and transparency of the selection process.

**FIGURE 2:**
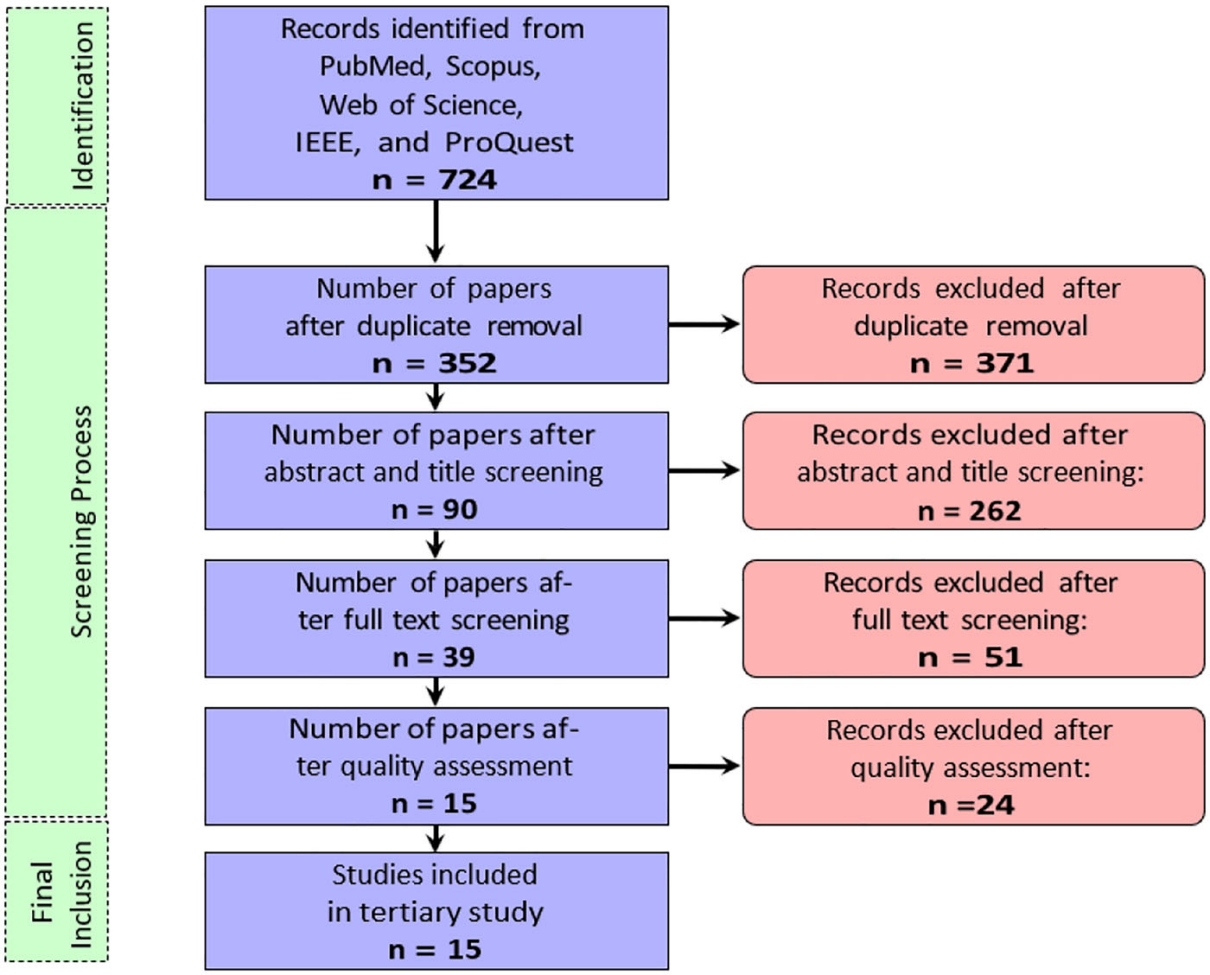
Flowchart illustrating the process of secondary study selection.

The screening phase began with the application of EC5 (Table 2) for deduplication, leaving 352 articles for preliminary title and abstract screening. During this process, the selection was conducted conservatively to mitigate the risk of false negatives. Consequently, papers were rejected only if they did not constitute a literature review (EC1) or were clearly out of scope (EC2) (Goulão et al. 2016). Following this stage, 262 papers were excluded. The subsequent step entailed a comprehensive full‐text review and, where feasible, data extraction of the remaining 91 secondary studies. At this stage, additional studies were excluded based on language constraints (EC4) or because they failed to identify primary studies employing algorithms for FS‐related issues (EC3). Figure 2 illustrates an overview of the previous and next filtering steps and the number of reviews retained at each stage.

### 3.4 Quality Assessment

In this tertiary study, the Database of Abstracts of Reviews of Effects (DARE) criteria proposed by the Center for Reviews and Dissemination (CDR) at York University were applied. These criteria have been widely utilized in various fields, including software engineering (Goulão et al. 2016; Ligthart et al. 2021) and consist of four critical questions (CQs) and a standardized scoring procedure. Each selected secondary study was scored based on a three‐point scale: “yes” (1), “partial” (0.5), and “no” (0) (Table 3).

**TABLE 3:** Quality criteria questions and scoring metrics.

Quality criteria questions	Scoring metrics
CQ1: Are reviews' inclusion and exclusion criteria described and appropriate?	Y (yes), the inclusion criteria are explicitly defined in the paper, P (partly), the inclusion criteria are implicit; N (no), the inclusion criteria are not defined and cannot be readily inferred
CQ2: Is the literature search likely to have covered all the relevant studies?	Y, the authors have either searched four or more digital libraries and included additional search strategies, such as snowballing, or identified and referenced all journals addressing the topic of interest; P, the study reports on using 3 or 4 digital libraries with no extra search strategies or searched a defined but restricted set of journals and conference proceedings; N, if previous two conditions were not fulfilled. Note that scoring question 2 also requires the evaluator to consider whether the digital libraries were appropriate for the specific SLR
CQ3: Did the reviewers assess the quality/validity of the included studies?	Y, if quality criteria were explicitly defined and extracted from each primary study; P, the research question involves quality issues that are addressed by the study; N no explicit quality assessment of individual papers has been included
CQ4: Were the basic data/studies adequately described?	Y, information is presented about each paper so that the data summaries can clearly be traced to relevant papers; P, only summary information is presented about individual papers. For example, papers are grouped into categories, but it is not explicit which papers belong to each category; N, the results of the individual studies are not specified. For example, the primary studies are not cited

A significant proportion of the reviewed papers lacked a clearly defined research methodology, and in several instances, the methodological details were minimal or absent. As a result, to ensure the integrity of the synthesis, only studies that exhibited a minimum level of methodological transparency were retained (i.e., a quality score > 0).

### 3.5 Information Extraction

For each 1 of the 15 selected reviews, information regarding the type of food or food category, data sources, the specific FS hazard, and AI algorithms was manually extracted. Because the scope of the secondary reviews was occasionally broad and included topics such as FF or FQ, only examples of FS were considered.

To address RQ2.1, distinct food categories were established based on the extracted data, and the frequency with which each category was addressed across the reviews was quantified. A similar process was followed for RQ2.2; however, in this instance, the specific secondary studies from which the data originated were explicitly identified.

For RQ2.3 and RQ2.4, primary studies referenced within the selected reviews that utilized specific algorithms to address FS‐related issues were extracted, resulting in a total of 190 unique instances. These instances were subsequently classified according to the type of FS hazard and the specific AI algorithm architecture employed.

### 3.6 Bibliometric Networks and Additional Data

To further examine the scope of the selected articles, a term co‐occurrence analysis was performed using bibliometric network methods. Overlay visualizations of these networks were generated using *VOSviewer*, an open‐access software specifically developed for constructing and exploring bibliometric maps (van Eck and Waltman 2017). A network visualization was developed encompassing terms that were mentioned at least four times within the titles and abstracts. In these networks, nodes represent elements such as authors or keywords; node size reflects their relative frequency or prominence, while colors denote clusters of thematically related items (Bouzembrak et al. 2019).

Table 4 provides an overview of the final 15 selected secondary studies, categorized by research scope, number of primary studies analyzed, publication year, and data sources. These studies form the foundational basis for the synthesis and analysis presented in the subsequent sections.

**TABLE 4:** Data extracted from the 15 secondary studies selected. NM, not mentioned.

Reference	Title	Studies included	Year	Source
Abid et al. (2024)	Quantitative and qualitative approach for accessing and predicting food safety using various web‐based tools	NM	2024	Google Scholar
Aggarwal et al. (2024)	Detection of mycotoxin contamination in foods using artificial intelligence: A review	9302 total	2024	Google Scholar
Moulahoum and Ghorbanizamani (2024)	Navigating the development of silver nanoparticles based food analysis through the power of artificial intelligence	120	2024	Web of Science
Inglis et al. (2024)	Machine learning applied to the detection of mycotoxin in food: a systematic review	30	2024	Scopus
Onyeaka et al. (2024)	Advancing food security: The role of machine learning in pathogen detection	NM	2024	PubMed, IEEE Xplore, Scopus, Web of Science, and Google Scholar
Z. Liu et al. (2023)	Artificial intelligence in food safety: A decade review and bibliometric analysis	28	2023	Web of Science
Pérez‐Santín et al. (2023)	Applicability domains of neural networks for toxicity prediction	12	2023	NM
Yuvaraj et al. (2023)	Implementation of information and communication technologies in fruit and vegetable supply chain: A systematic literature review	99	2023	Scopus, Web of Science, ScienceDirect
Chen et al. (2023)	Review of visual analytics methods for food safety risks.	More than 100	2023	NM
Gul et al. (2024)	Deep learning hyperspectral imaging: A rapid and reliable alternative to conventional techniques in the testing of food quality and safety	305 total	2024	Web of Science, Scopus, Google Scholar
Raki et al. (2024)	Combining AI tools with non‐destructive technologies for crop‐based food safety: A comprehensive review	69	2024	Web of Science, Scopus, IEEE Xplore
Mu et al. (2024)	Making food systems more resilient to food risks by including artificial intelligence, big data, and internet of things into food safety early warning and emerging risk identification tools	40 Articles + 57 reviews	2024	Scopus, ScienceDirect, Google Scholar
Kim and Kim (2022)	Impact and prospect of the fourth industrial revolution in food safety: Mini‐review	NM	2022	Google Scholar
Wang et al. (2022)	Application of machine learning to the monitoring and prediction of food safety: A review	114	2022	Scopus, CAB Abstracts, IEEE
Talari et al. (2022)	State of the art review of big data and web‐based decision support systems (DSS) for food safety risk assessment with respect to climate change	92	2022	Web of Science, Scopus, ScienceDirect, Google Scholar

## 4 Results

In this section, the findings synthesized from the 15 selected secondary studies are presented. The information is organized thematically, with subsections corresponding to each research question.

### 4.1 RQ1.1: What Research Methodologies Are Employed in the Analyzed Reviews?

Among the 15 selected secondary studies, a significant portion exhibited poorly defined research procedures with limited methodological transparency, often limited to a brief description of keywords and sources. Consequently, the quality assessment of these articles, including both the final candidates and those excluded during the screening phase‐resulted in generally low scores. Table 5 summarizes the methodological completeness of the 15 final reviews.

**TABLE 5:** An overview of some key research methodological components extracted from the 15 selected reviews, highlighting common elements such as inclusion and exclusion (I/E) criteria and quality assessment (QA).

Review	Keywords	Studies included	Sources	Date range	I/E criteria	QA	Protocol
Abid et al. (2024)			×				
Aggarwal et al. (2024)	×	×	×	×			(Khan et al. 2022)
Moulahoum and Ghorbanizamani (2024)	×	×	×	×			
Inglis et al. (2024)	×	×	×	×			
Onyeaka et al. (2024)	×		×	×	×		
Z. Liu et al. (2023)	×	×	×	×			
Pérez‐Santín et al. (2023)	×	×		×	××		
Yuvaraj et al. (2023)	×	×	×	×	×	×	SLR
Chen et al. (2023)		×		×			
Gul et al. (2024)		×	×				PRISMA
Raki et al. (2024)	×	×	×	×	×		own
Mu et al. (2024)	×	×	×	×			PRISMA
Kim and Kim (2022)	×		×	×			
Wang et al. (2022)	×	×	×	×	×		EFSA Guidance for those carrying out systematic reviews European Food Safety Authority (2010)
Talari et al. (2022)	×	×	×	×			

Abbreviation: PRISMA, Preferred Reporting Items for Systematic Reviews and Meta‐Analyses.

The majority of the reviewed articles demonstrated a notable lack of adherence to formal research methodologies or standardized protocols. Furthermore, the description of the literature retrieval and analysis was frequently confined to one or two brief paragraphs, lacking the detail necessary for reproducibility. In the instance of Mu et al. (2024), the research methodology was relegated to the Supporting Information rather than being integrated into the main text; in addition, this study adopted a hybrid approach by reviewing both primary and secondary studies.

Among the analyzed secondary studies, only six reviews documented the protocol employed. While Yuvaraj et al. (2023) and Gurbuz and Tekinerdogan (2018) explicitly referenced established protocols, other studies asserted adherence to systematic approaches without specifying a formal framework (Wang et al. 2022). Alternative approaches identified in the literature included citing an external reference as a methodological guide (Aggarwal et al. 2024) or employing proprietary frameworks, such as “TriScope Keywords‐based Synthesis,” which the authors suggest can be generalized to a “MultiScope Keywords‐based Synthesis” (Raki et al. 2024).

In addition, certain reviews employed advanced analytical techniques such as clustering, following the initial article selection phase. For instance, Kim and Kim (2022) utilized clustering to thematically visualize analyzed data, while Talari et al. (2022) categorized their studies into five clusters derived from bibliographic network analysis. Furthermore, Z. Liu et al. (2023) conducted a research hotspot analysis utilizing CiteSpace; similarly, Yuvaraj et al. (2023) employed VOSviewer to generate the bibliographic network visualizations based on author‐supplied keywords.

### 4.2 RQ1.2: What Is the Scope of the Reviews?

**FIGURE 3:**
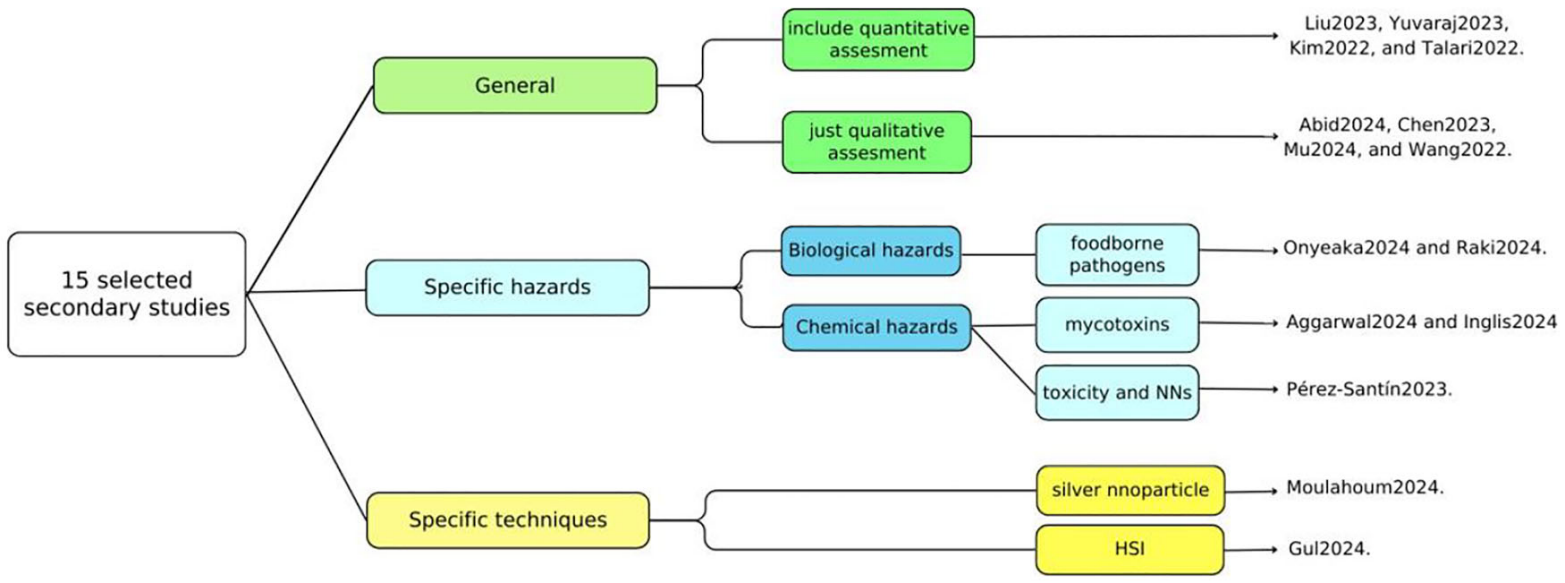
Categorization structure of the relevant secondary studies based on their scope. (a) Keyword network visualization illustrating four different clusters. (b) Time distribution network visualization.

As depicted in Figure 3, the thematic scope of the selected reviews can be categorized into three distinct domains: General perspectives, specific hazards, and specific techniques. The first category encompasses secondary studies that adopt a holistic or general perspective regarding AI applications within the broader context of FS. For example, Z. Liu et al. (2023) explored AI in FS through a bibliometric methodology, offering comprehensive insights into the bibliographic metadata of the primary studies included in the review. Similarly, Kim and Kim (2022) examined the impacts of Industry 4.0 paradigms on FS; their work elucidated the general methodological process of model development and provided illustrative examples of ML applications across the food industry.

The subsequent category focuses on secondary studies that address specific FS hazards possessing the potential to risk consumer health, classified into biological, chemical, and physical FS hazards. For instance, Aggarwal et al. (2024) and Inglis et al. (2024) investigated the detection of mycotoxin contamination in foods using AI, addressing a key chemical hazard. Meanwhile, Onyeaka et al. (2024) reviewed the application of ML methods for the identification of foodborne pathogens, focusing on biological threats.

The final category included secondary studies focused on specific techniques employed for FS hazard detection, specifically HSI and silver nanoparticle (AgNP) sensors. While HSI data was mentioned in numerous other reviews, Gul et al. (2024) provided a detailed introduction to its technical fundamentals and elucidated the basic DL model architectures utilized within the food sector. In the domain of FS, this technique is primarily applied to the detection of microbial and toxicant contamination; however, it also possesses application in sensory analysis or assessment of physicochemical properties.

The second review in this category concentrated on the application of AgNPs in FS for detecting contaminants, including heavy metals, pesticides, and pathogens, even at low concentrations. Moulahoum and Ghorbanizamani (2024) presented these as innovative alternatives to traditional, established methods such as spectrophotometry and chromatography. The review highlighted the potential of integrating AgNPs as sensing probes with methodologies including colorimetric sensing, immunoassay, surface‐enhanced Raman spectroscopy (SERS), and electrochemical techniques.

While the majority of the studies adopted a generalized approach, several addressed significant peripheral themes. For instance, Wang et al. (2022) investigated the influence of climate change on FS predictive modeling, highlighting how ML can integrate meteorological shifts and nonlinear environmental variables to forecast long‐term risks, such as aflatoxin prevalence. Yuvaraj et al. (2023) focused on the implementation of Information and Communication Technologies (ICTs) within specialized sectors, such as the fruits and vegetables supply chain. By utilizing IoT sensors and NNs for real‐time monitoring of temperature and ethylene levels, this approach emphasized immediate operational control and the mitigation of “temperature excursions” to ensure safety and quality. Furthermore, a significant proportion of the selected studies reviewed primary literature that integrated FS issues with related domains, including FQ, FF, or traceability. Consequently, to maintain the thematic integrity of this study, the following sections will exclusively address findings and examples pertaining to FS.

#### 4.2.1 Bibliometric Networks

**FIGURE 4:**
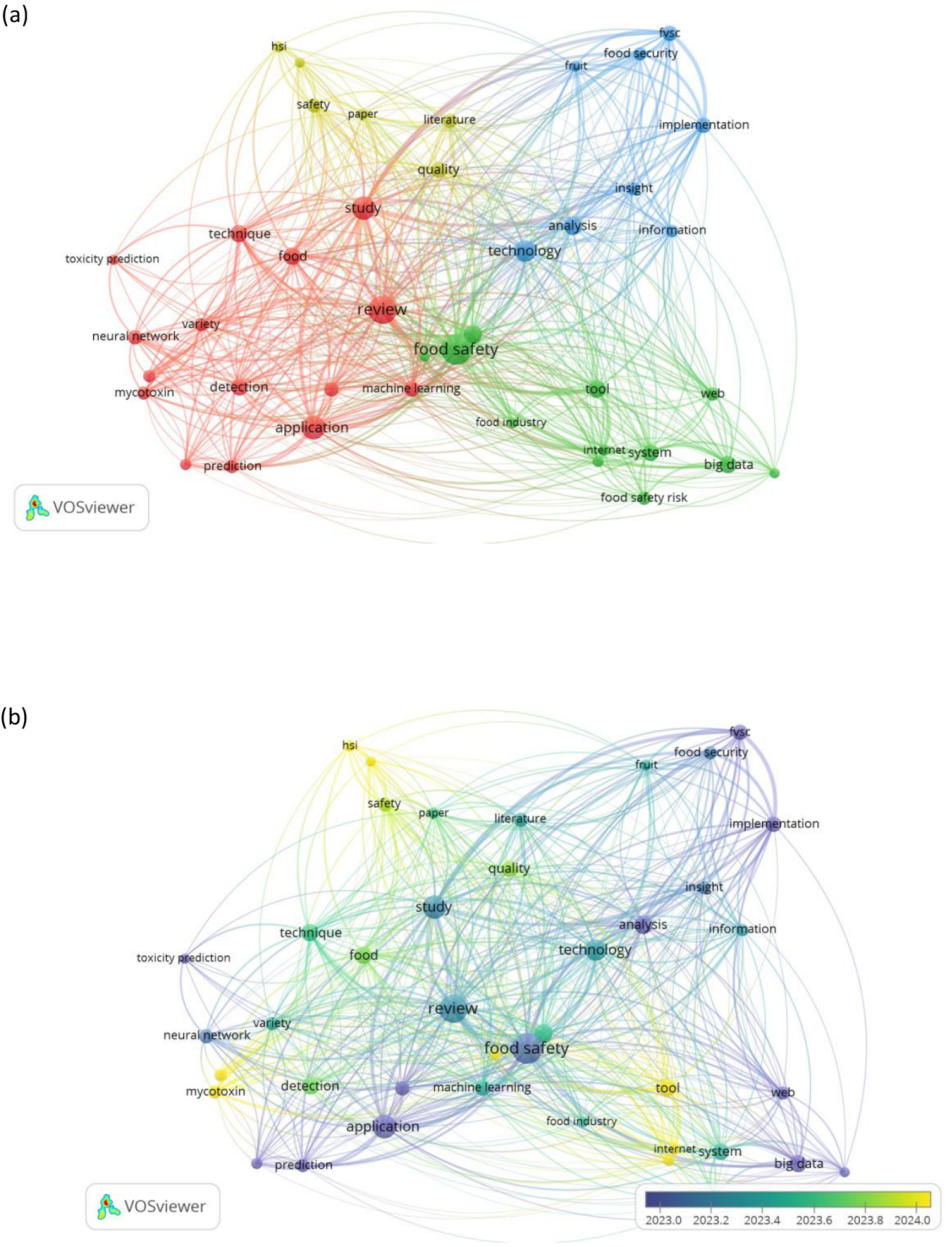
Visualization of keyword co‐occurrence from titles and abstracts using VOSviewer, highlighting the most frequently mentioned terms and their relationships.

Figure 4 presents a network visualization of terms occurring at least four times within the titles and abstracts of the analyzed literature.

In Figure 4a, four different clusters emerged, representing various research focal points. One prominent cluster (i.e., green) revolves around big data, FS risks, and information, highlighting the role of data‐driven approaches and sensor‐based monitoring systems (IoT) in ensuring FS. The red cluster aggregated several applications, ranging from general detection and prediction techniques to specialized areas such as mycotoxin detection or toxicity prediction. The third cluster (i.e., yellow) included terms such as HSI, DL, and quality, indicating that a significant number of articles considered HSI a promising technique for simultaneous FS and quality assessment. As shown in Figure 4b, these terms were also highlighted in yellow on the temporal overlay, suggesting that they had been the focus of the most recent reviews. The final cluster (i.e., blue) comprised terms such as technology and information, likely referring to ICT paradigms, alongside food security and the fruits and vegetables supply chain. This reflects the extensive use of new technologies to enhance food security through optimized supply chain management.

### 4.3 RQ2.1: To Which Specific Foods or Products Has AI Been Most Frequently Applied to in FS?

The majority of the analyzed secondary studies did not focus on a specific food item; instead, a broad array of food types and supply chain stages was examined within the included primary literature. Notably, only one study was identified that specifically investigated the fruits and vegetables sector (Yuvaraj et al. 2023). Within this review, it was highlighted that fruit tended to attract greater research interest compared to other categories. Among these, apples, strawberries, grapes, and avocados were particularly emphasized. Furthermore, it was underscored by the authors that, despite this focus, the fruit and vegetables remained underexplored relative to their importance in FS.

**FIGURE 5:**
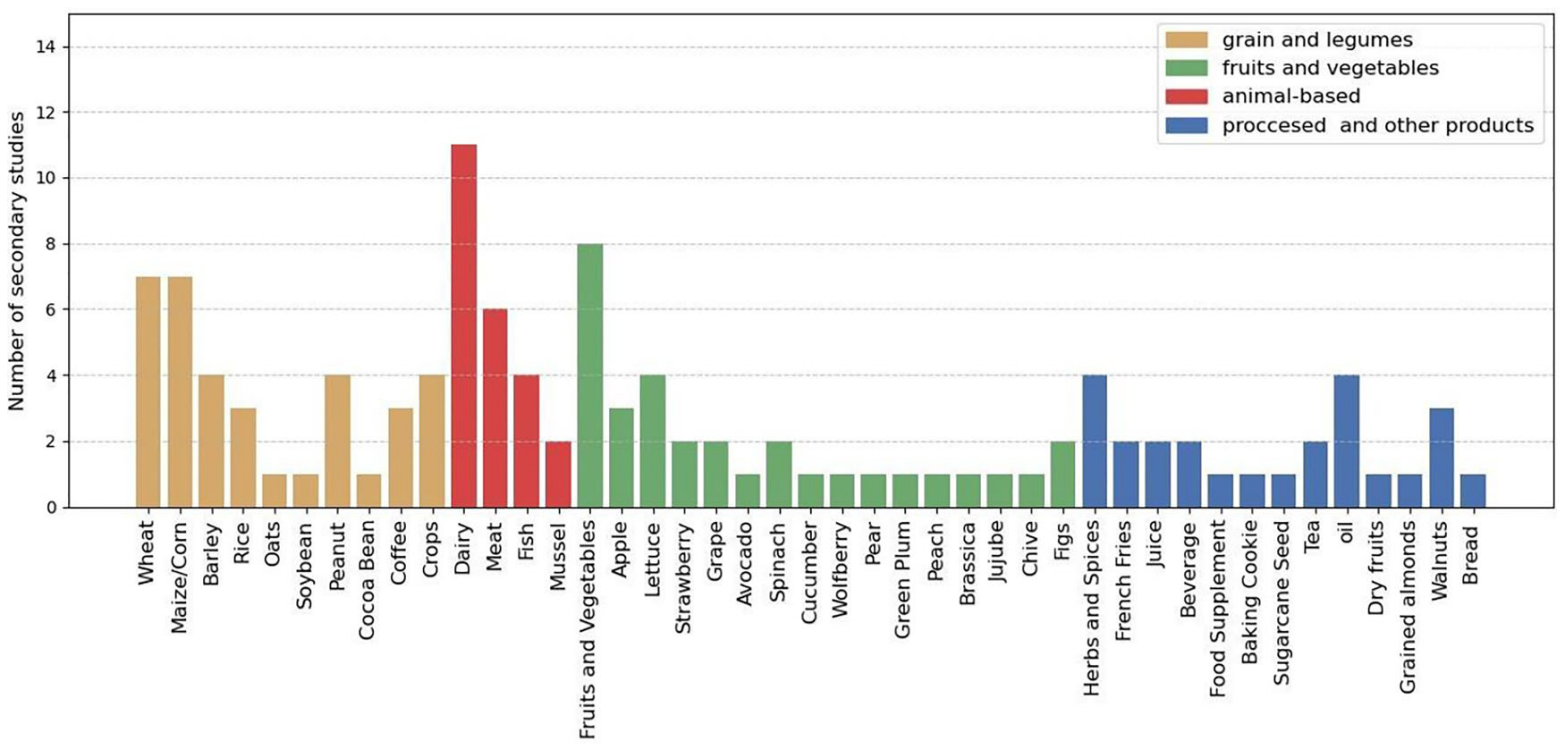
Foods or food categories terms and in how many out of the 15 selected secondary studies it has been mentioned, paired with FS and AI techniques.

An analysis of food‐related terms associated with FS issues, ranging from broad categories such as fruits and vegetables to highly specific items like French fries, was performed across the 15 selected articles. As was demonstrated in Figure 5, and contrary to the findings presented in Yuvaraj et al. (2023), fruits and vegetables emerged as a dominant research focus in FS research.

Dairy products were identified in 11 selected reviews, underscoring their significance in FS research. The scope of the studies was found to be extensive, covering diverse aspects of contamination and risk assessment. For instance, the detection of antibiotics, such as kanamycin, was explored utilizing nano‐biosensors (Pérez‐Santín et al. 2023). Foodborne pathogen detection was also examined, with specific research addressing the identification of *Listeria* (Onyeaka et al. 2024). Furthermore, the detection of adulterants detection was investigated using hyperspectral radiometry coupled with ML techniques, enabling the identification of contaminants such as soda, water, urea, and detergents (Gul et al. 2024). Beyond direct contaminant detection, research was directed toward broader risk predictions; this included the analysis of food inspection data in sterilized milk (Abid et al. 2024) and the assessment of climate impacts on FS hazards within the dairy supply chain (Talari et al. 2022). These findings highlighted the critical role of environmental and inspection data in ensuring safety in dairy products. In addition, crops were extensively addressed, with wheat and maize/corn emerging as the most frequently discussed commodities.

Within these food categories, certain primary studies were referenced in many reviews, contributing significantly to the frequency counts presented in Figure 5. These included research on the occurrence of chemical FS hazards in fruits and vegetables (Bouzembrak and Marvin 2019) and the development of an automated FS early warning system in the dairy supply chain (N. Liu et al. 2022).

Although most reviews did not address specific food items, the study of Mu et al. (2024) was notable for containing two detailed case studies regarding early warning systems for FS hazards. The first example was focused on harmful algae blooms, where biotoxins were found to affect aquatic organisms such as fish, mollusks, and crustaceans. Among these, mussels were identified as the most frequently consumed by humans, presenting a significant risk of illness. The second case study pertained to aflatoxin prediction in maize, pistachios, peanuts, and wheat. This predictive modeling was dependent upon diverse input parameters, including temperature, rainfall, wind speed, soil temperature and moisture, and crop phenology (Mu et al. 2024).

### 4.4 RQ2.2: What Types of Data Have Been Utilized Alongside AI in the Field of FS?

Regarding the data type identified, a categorization was established based on the different formats presented in literature: Sensing data were found to consist primarily of structured numerical values. Data retrieved from FS databases included both structured historical records and unstructured information, such as food alert reports. In addition, unstructured data were found to encompass both image‐based formats, such as HSI and textual data.

Sensing data were derived from sensors, spectroscopy, and other analytical detection methods such as chromatography or genomic analysis. Parameters, including temperature, moisture content, CO_2_ levels, and pH, were detected by sensors, which were typically acquired in real‐time via IoT technologies. These electro‐analytical methods were often integrated with spectral techniques for real‐time detection and higher accuracy results (Raki et al. 2024). An example of its application included the evaluation of the shelf life of wheat and barley‐based food through the detection of grain off‐odor caused by microbial contamination (Raki et al. 2024).

Spectroscopy techniques were further categorized based on the wavelength ranges measured by the spectrometer. Each range was found to offer distinct insights into the sample characteristics, rendering them suitable for specific food applications. Near‐infrared (NIR) spectroscopy was applied to detect DON in oat samples collected in Spain and Sweden (Inglis et al. 2024). Similarly, SERS was utilized to predict aflatoxin B1 levels in pressed peanut oil (Aggarwal et al. 2024). Fourier transform infrared (FTIR) spectroscopy facilitated the detailed characterization of food components and contaminants; it was employed to detect aflatoxin contamination in figs (Raki et al. 2024). Furthermore, fluorescence spectroscopy, which measures the fluorescence emissions of a sample, was used to identify aflatoxins B in ground almonds (Raki et al. 2024).

In addition, nuclear magnetic resonance (NMR) was found to provide comprehensive molecular‐level information. Although less commonly applied in FS, it was utilized to distinguish between microbial strains in food‐based metabolite profiles (Raki et al. 2024). Chromatographic techniques such as liquid chromatography–mass spectrometry (LC–MS), high‐performance liquid chromatography (HPLC), and gas chromatography–mass spectrometry (GC–MS), along with immunoassay‐based methods like enzyme‐linked immunosorbent assays (ELISAs) were traditionally employed for mycotoxin detection.

However, these techniques were observed to have evolved toward nondestructive techniques in recent years. For instance, the combination of thin‐layer chromatography (TLC) with SERS was implemented for histamine analysis in tuna (Pérez‐Santín et al. 2023). Finally, regarding genomic analysis, techniques such as next‐generation sequencing (NGS) and PCR were used to generate genomic data. This information was subsequently employed to effectively predict the food source of clinical *Listeria monocytogenes* (Onyeaka et al. 2024).

Data from online FS databases referred to large‐scale datasets that provided contextual insights into FS and supply chain analysis. These sources encompassed weather data, crop data, environmental data, economic data, or FS alerts extracted from different open‐access databases like Rapid Alert System for Food and Feed (RASFF) or the Royal Netherlands Meteorological Institute (KNMI). These datasets were applied to predict the risk of crop contamination and to identify vulnerabilities within the food supply chain (Abid et al. 2024).

Image data, which consisted of visual information, was typically captured in 2D formats. This type of data was used for detecting physical FS hazards and assessing product quality. Examples encompassed satellite images for monitoring algal blooms (Mu et al. 2024), food images, and UV‐captured pictures. HSI was found to occupy a hybrid space between chemical and image data, by simultaneously capturing surface and internal information in the food samples (Gul et al. 2024). This technique was widely employed for the precise identification of microbial contamination, pesticides, and heavy metals. Similar to spectroscopy methods, HSI was classified based on the wavelength ranges; common modalities identified included multispectral imaging, visible HSI (VIS), visible NIR HSI (VNIR), fluorescence imaging, Raman imaging, and terahertz (THz) spectral imaging.

Specific applications were documented, such as the use of HSI to detect *Escherichia coli* count in grass carp (Gul et al. 2024), and pesticides in apples. In addition, VNIR was utilized for the enhanced prediction of lead and cadmium content in lettuce (Pérez‐Santín et al. 2023).

Text data were categorized as unstructured textual data retrieved from diverse sources, including social media, customer reviews, academic publications, and regulatory documents. This information was analyzed to detect emerging FS risks or public concerns. One notable example was a study conducted using Twitter as input for a text mining ML model; their research demonstrated a significant correlation with the actual incidence of romaine lettuce food poisoning in the USA in 2018 (Mu et al. 2024).

**FIGURE 6:**
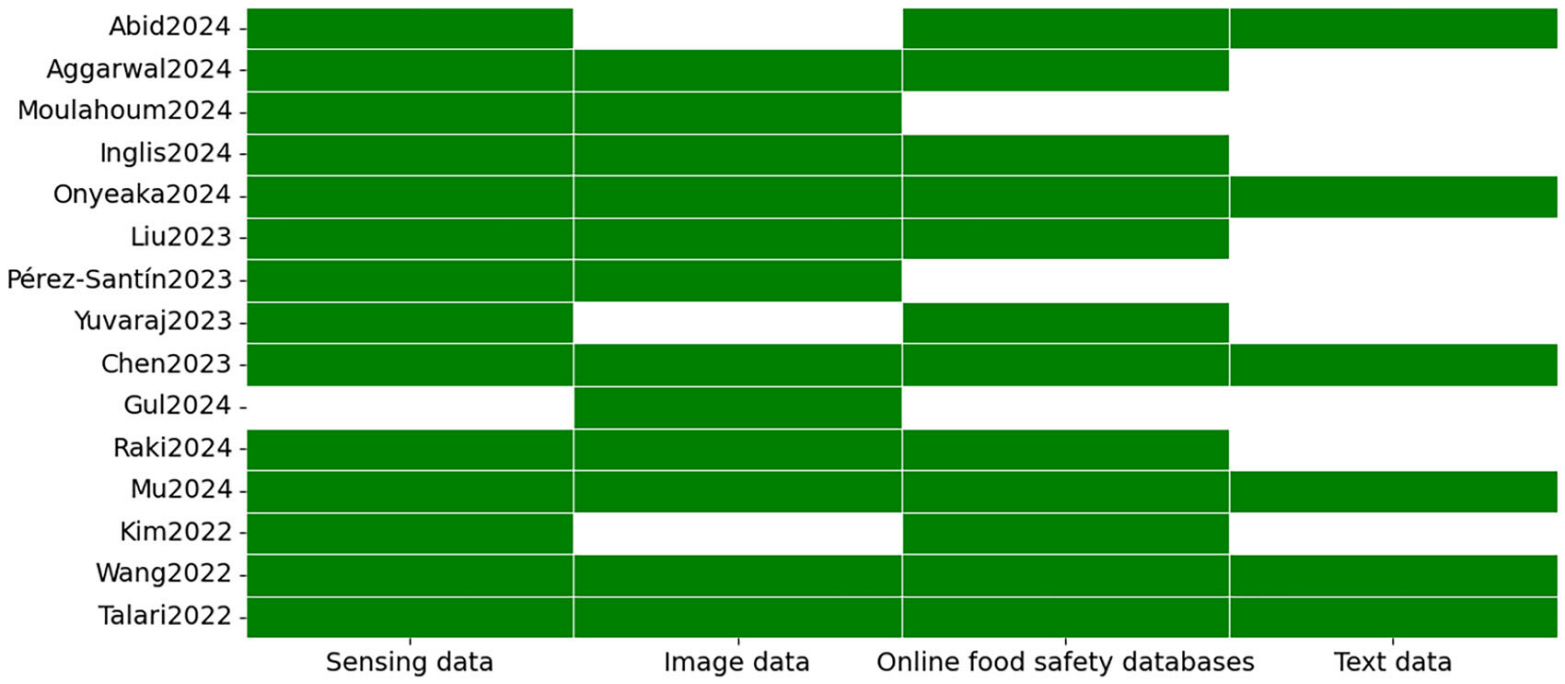
Graph summarizing the type of data included in each selected review, on the *x*‐axis the different data categories, and on the *y*‐axis the reference to each selected study.

As was illustrated in Figure 6, with the exception of Gul et al. (2024), which exclusively addressed “image data” due to its specific focus on HSI, the remaining selected articles reviewed a variety of data types. “text data” was addressed in six reviews, whereas “sensing data” was identified in fourteen of the 15 selected studies. This provided significant insights into the current state of FS modeling, which was found to rely more on empirical, quantitative data than on textual sources that offered more qualitative and interpretative information. Furthermore, while “online FS databases” were cited in 12 of the selected articles, only 6 explicitly documented the specific open‐source databases from which the data were extracted.

**FIGURE 7:**
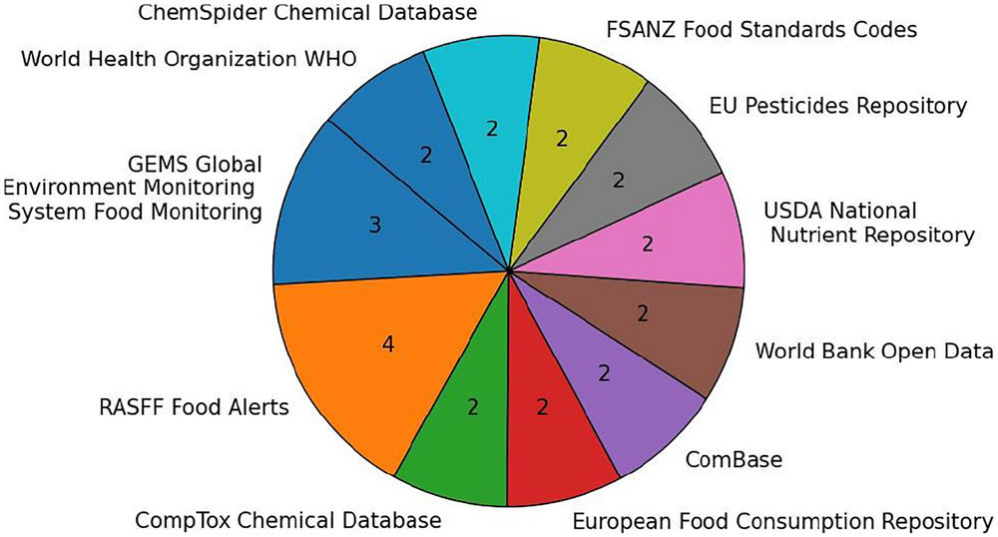
The 11 databases that are mentioned in two or more selected reviews.

As was depicted in Figure 7, the most frequently referenced FS databases were the RASFF for alert notifications, the Global Environment Monitoring System (GEMS) for data monitoring, and the European Food Consumption Repository (Abid et al. 2024). Other studies also mentioned the EU Pesticide Repository or the World Bank Open Data. All databases identified within the selected secondary studies were consolidated in Table S7.

### 4.5 RQ2.3: Which Specific FS Hazards Have Been Addressed Using AI?

**FIGURE 8:**
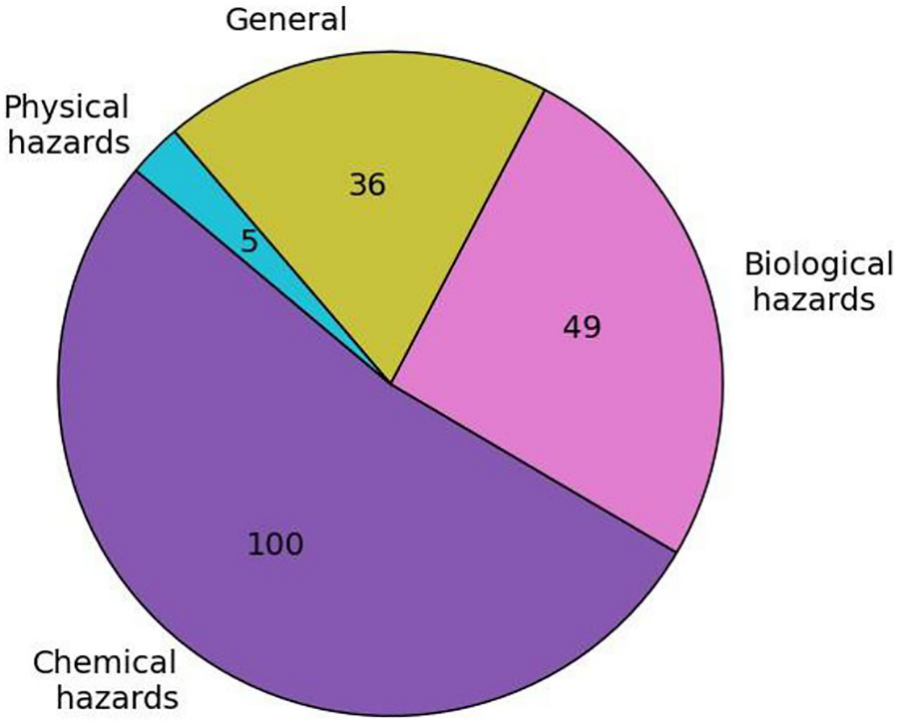
Frequency of chemical, biological, physical, and general hazards identified in the 190 examples extracted from the selected reviews.

Each of the 15 selected reviews, primary studies that specified algorithms to address an FS‐related issue, was extracted, resulting in a total of 190 instances. Figure 8 illustrates that chemical FS hazards constituted the majority of reported cases, whereas physical hazards were found to be the least frequently addressed.

Chemical FS hazards were documented in the literature 100 times (Figure 8), encompassing the identification and detection of toxins, such as mycotoxins and pesticide residues, as well as the prediction of heavy metals presence (Wang et al. 2022). One of the most referenced compounds was aflatoxin, a toxic chemical produced by certain molds that commonly affect food products susceptible to fungal growth, including corn, peanuts, spices, and figs (Inglis et al. 2024).

Regarding biological FS hazards, the research conducted by Onyeaka et al. (2024) and Raki et al. (2024) was focused on the detection of foodborne pathogens. In the study by Raki et al. (2024), detection methods for aflatoxin were also included; it was noted that while these are primarily considered as chemical FS hazards, they were also utilized to address microbial risks and the presence of specific molds, such as *Aspergillus flavus* and *Aspergillus parasiticus*. In the review by Onyeaka et al. (2024), several instances of foodborne pathogen detection were documented, including *Pseudomonas aeruginosa*, *Salmonella*, and *E. coli*. Furthermore, a model was presented for forecasting the spatiotemporal nature of salmonellosis in Italy, using data retrieved from FS audits.

Physical FS hazards were less frequently addressed in the literature. Among the few examples identified, foreign object identification in walnuts was performed using extreme learning machine (ELM) and support vector machine (SVM) (Z. Liu et al. 2023) or implemented through using CNNs (Wang et al. 2022). These studies were closely linked to CV techniques, where images were used as primary input data.

Finally, a subset of primary studies adopted a broad approach toward FS. These studies, which were capable of utilizing many different types of data to identify risks associated with FS hazards, were referred to as “general” (Wang et al. 2022). Notable examples included research focused on the development of early warning systems, the prediction of several FS hazards, the automation of FS inspections, and the implementation of food monitoring systems.

### 4.6 RQ2.4: Which AI Algorithms or Models Are Commonly Used in FS‐Related Problems?

**FIGURE 9:**
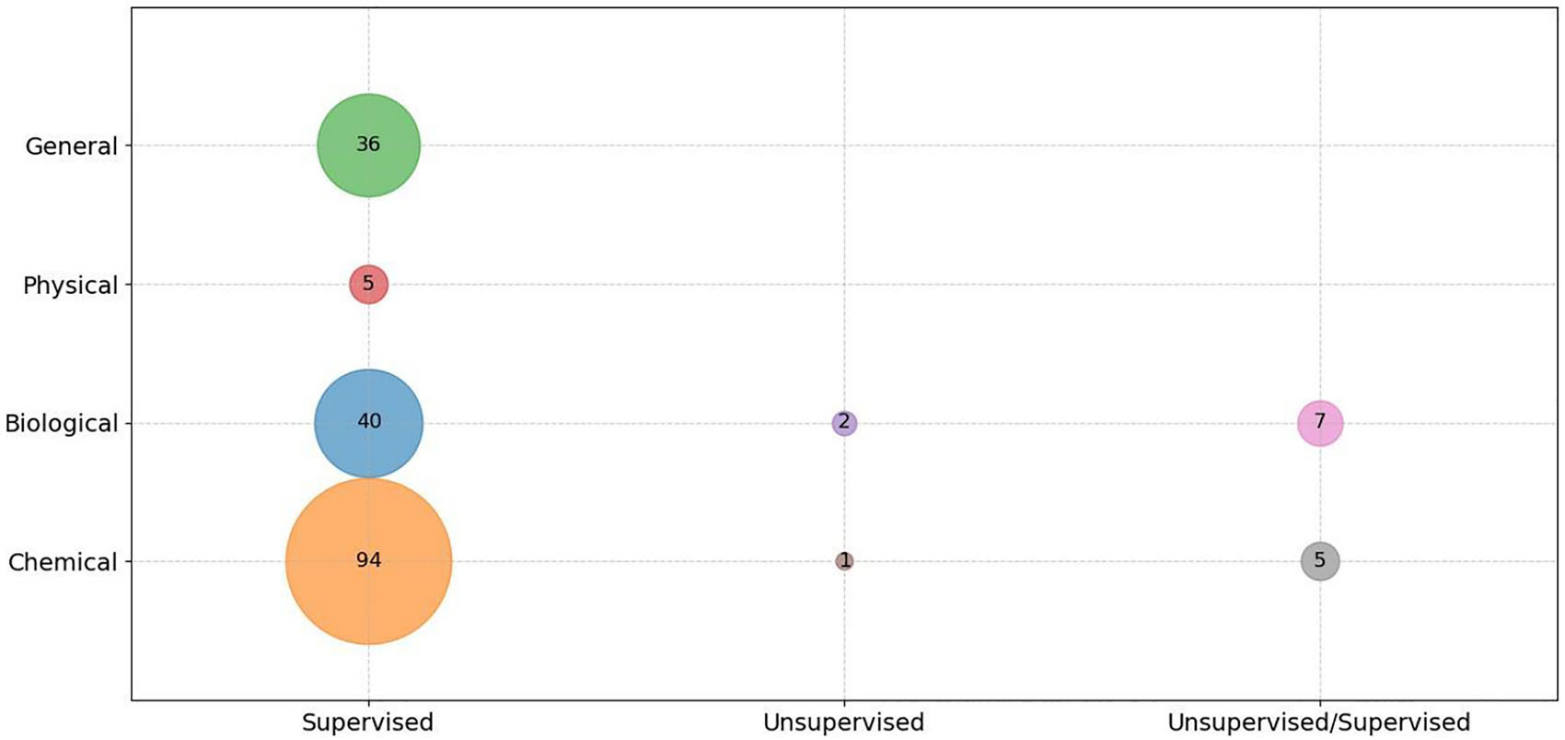
Bubble graph illustrating the relationship between supervised, unsupervised and unsupervised/supervised (*x*‐axis), and different types of FS hazards (*y*‐axis). The size of the bubbles corresponds to the number of examples that establish a specific connection.

The algorithms identified in the selected articles were first categorized as “supervised,” “unsupervised,” and “unsupervised/supervised.” The findings were summarized in Figure 9, which illustrated the distribution of these categories across different types of FS hazards. The primary and most frequently utilized algorithms were identified as “supervised” methods, which facilitate learning from labeled datasets. They were employed for both classification and regression tasks, prominent examples encompass SVM, NNs, or Decision Trees (DTs).

In contrast, unsupervised methods rely on unlabeled data and are commonly used for clustering and dimensionality reduction. Popular unsupervised methods included principal component analysis (PCA), autoencoders, *K*‐means clustering, and hierarchical clustering. Finally, in instances where both categories were explicitly mentioned within a single study, the “unsupervised/supervised” classification was applied. These examples primarily referred to studies that, despite having labeled data, also performed some form of data pre‐analysis using unsupervised methods.

**FIGURE 10:**
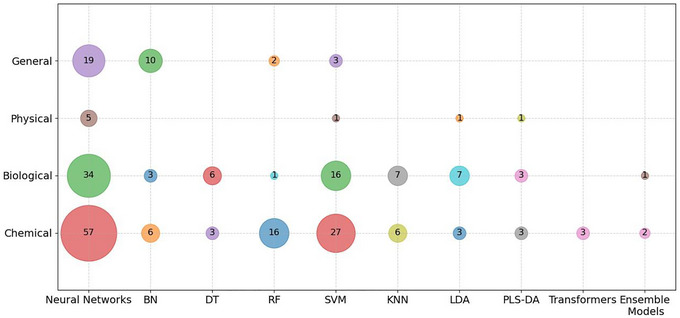
Bubble graph illustrating the relationship between different algorithms (*x*‐ axis) and different types of FS hazards (*y*‐axis). The size of the bubbles corresponds to the number of examples that establish a specific connection.

**FIGURE 11:**
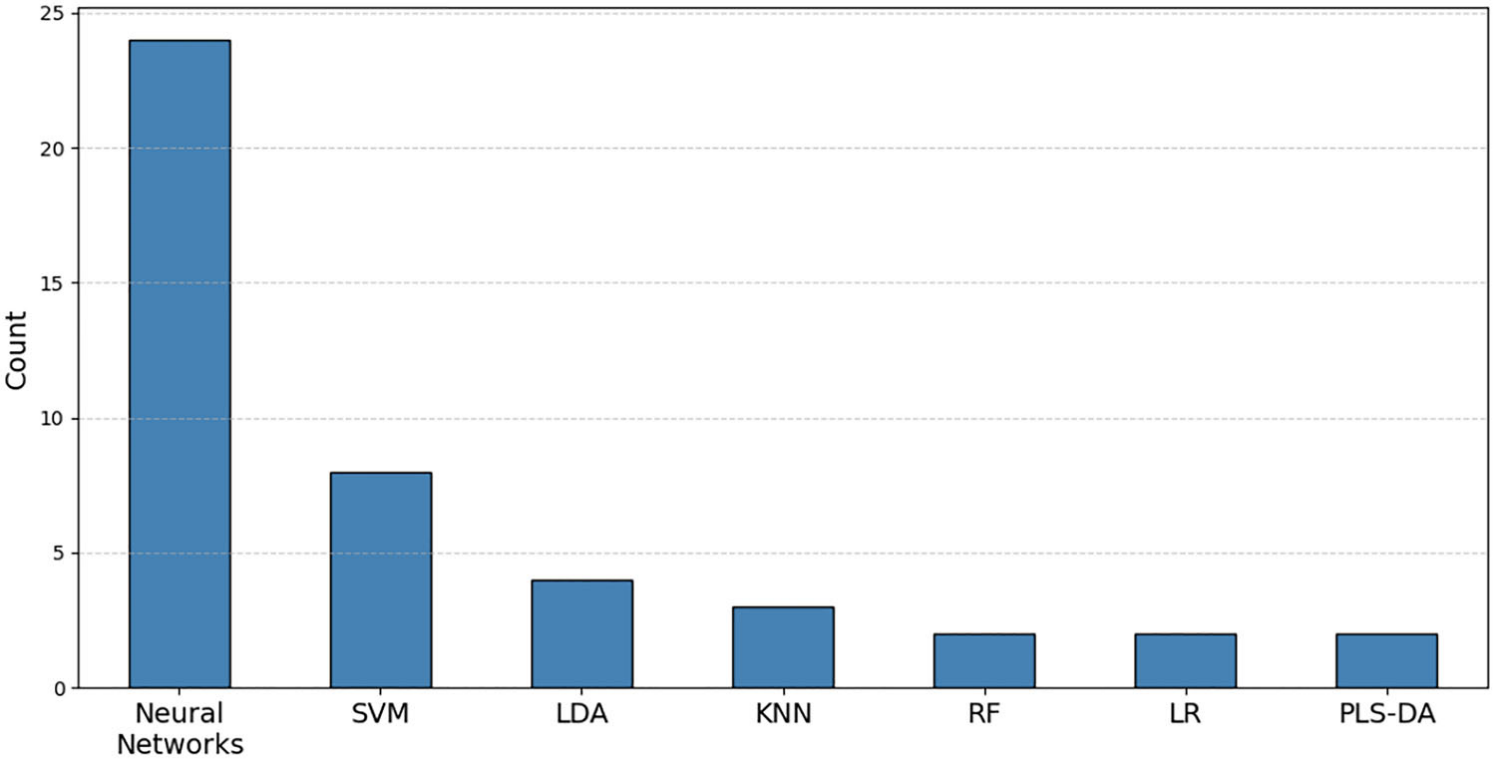
Seven most used algorithms in HSI.

To better understand the diverse applicability of the algorithms utilized within FS, a more extensive data analysis was performed. Every algorithm, including instances where multiple models were compared with, belonging to the “supervised” and “unsupervised/supervised” categories, was counted and annotated according to the specific FS hazard it addressed. This process yielded 312 instances, which were grouped based on the algorithm type and the associated hazard. As was illustrated in Figures 10 and 11, the most frequently cited algorithms included: NNs, Bayesian networks (BN), DT, Random Forest (RF), SVM, *K*‐nearest neighbors (KNN), linear discriminant analysis (LDA), partial least squares discriminant analysis (PLS‐DA), transformers and ensemble models were identified as significant contributors to the current FS research landscape.

The results highlighted that NNs were predominantly used to address chemical hazards. This finding aligned with the observations by Pérez‐Santín et al. (2023), who emphasized the significance of NNs in chemical FS hazards prediction, and by Inglis et al. (2024), who specifically identified CNNs as the preferred and most frequently employed technique for mycotoxin data analysis.

NNs attained widespread adoption in FS applications, particularly in HSI, due to their ability to handle the complexity of the spatial and spectral HSI data. As was illustrated in Figure 10, NNs were ranked as the primary algorithmic choice for this data type, followed by SVM.

Furthermore, NNs were frequently integrated with ML approaches such as RF, KNN, SVM, LDA, and PLS‐DA to enhance overall classification performance (Aggarwal et al. 2024). These combined approaches fall under the Ensemble category; a notable example was the SAE‐CNN‐SVM model, which was found to achieve the most satisfactory result in the grading of moldy maize kernels (Aggarwal et al. 2024). In this architecture, a sparse autoencoder (SAE) was utilized for feature extraction, while the CNN‐SVM components facilitated robust classification and decision‐making.

BNs models were identified as another prominent within the selected studies. These models excelled at analyzing variable dependencies, mapping nonlinear interactions, and incorporating diverse formats of structured data (Inglis et al. 2024). BNs were found to be particularly effective in managing the heterogeneous nature of FS data, rendering them suitable for environments with complex data structures where probabilistic relationships must be established among variables. Furthermore, this methodology facilitated the seamless incorporation of expert knowledge, thereby enhancing the reliability of the resulting predictive models (Mu et al. 2024).

Examples of BN applications across chemical, biological and general hazard were documented as follows: (i) chemical hazards, the prediction of pesticides and mycotoxins in fruits and vegetables across three geographically distinct countries or the prediction of deoxynivalenol (DON) levels in Dutch wheat utilizing incomplete datasets; (ii) biological hazards, the implementation of microbial risk assessments to evaluate pathogen proliferation (Talari et al. 2022); and (iii) general hazards, the prediction of the critical FS hazards and high‐risk products within herbs and spices sector, as well as the analysis of FS‐related traceability data to identify potential contamination points throughout food production process (Wang et al. 2022).

SVM and KNN were also found to be widely used in classification tasks. These algorithms were extensively applied across various domains such as sensing, spectroscopy, and classifying compounds. For instance, SVM was employed for the detection of microbial contamination in fruits and vegetables. Furthermore, it was used in combination with KNN to classify ambiguous *Salmonella* spp. contamination (Moulahoum and Ghorbanizamani 2024). The KNN algorithm was also utilized for classification tasks and forecasting of aflatoxin B1 level within maize kernels (Abid et al. 2024; Wang et al. 2022). Other specialized classification methods, including LDA, least squares SVM (LSVM), quadratic discriminant analysis (QDA), and quantum SVM (QSVM), were applied to detect microbial spoilage in lettuce (Raki et al. 2024).

Tree‐based models, such as DT and RF, were widely employed through the reviewed literature. It was particularly noteworthy to observe that RF was exclusively utilized in the context of general hazards; examples of application included risk prediction (Mu et al. 2024) and the classification of mussel origin to predict potential contamination levels (Wang et al. 2022). Conversely, DT was more frequently applied to biological FS hazards. Aside from the aforementioned general hazards examples, RF was predominantly used for chemical FS hazards. The underlying reason for this distribution lay in the complexity and structure of the data. Biological FS hazards often involve categorical classifications like the presence or absence of bacteria or the identification of foodborne pathogens. In these instances, DT is well‐suited due to its inherent simplicity and interpretability. In contrast, chemical FS hazards are often present in complex structures, where multiple factors influence contamination risk. Therefore, a model such as RF, which improves predictive accuracy and reduces overfitting by aggregating multiple DTs, was found to be preferable for these high‐dimensional chemical datasets.

Furthermore, validation metrics and methodologies were not consistently reported across the selected studies. While some articles provided accuracy scores or identified the most effective approach, particularly in the context of comparative analyses, many lacked detailed information about the validation procedures. The most frequently utilized validation techniques included random sampling, *k*‐fold cross‐validation, and bootstrapping (Wang et al. 2022). Internal validation was identified as the predominant method, wherein a specific portion of the dataset is reserved to evaluate model performance. Regarding performance metrics, accuracy was documented as the most frequently reported measure. In addition, in the review by Gul et al. (2024), specific hyperparameters unique to NNs, such as kernel size or number of filters, were included in the reporting.

### 4.7 RQ2.5: What Are the Key Challenges Regarding FS and AI?

All but one of the selected articles discussed challenges in the application of AI techniques to FS issues. Table 6 provides detailed information about these challenges, which were further sorted based on the total number of selected papers explicitly mentioning each challenge. Given the diverse nature of these challenges, they can be summarized as follows:

**TABLE 6:** Challenges and considerations for AI applications in FS.

Challenge	Description	Selected reviews	Count
Data quality and standardization	Include dealing with inconsistent formats (data structure, measurements, scales); not properly or poorly annotated data; data reliability; data fairness (FAIR principles); and standardization, uniform methodologies for data collection and validation	Aggarwal et al. (2024), Onyeaka et al. (2024), Pérez‐Santín et al. (2023), Mu et al. (2024), Moulahoum and Ghorbanizamani (2024), Abid et al. (2024), Inglis et al. (2024), Talari et al. (2022), Wang et al. (2022), and Raki et al. (2024)	10
Regulatory and ethical considerations	Legal compliance with regulations such as GDPR and OECD rules. Ethical issues in AI require responsibility, openness, and legal compliance	Aggarwal et al. (2024), Onyeaka et al. (2024), Pérez‐Santín et al. (2023), Mu et al. (2024), Kim and Kim (2022), Chen et al. (2023), and Moulahoum and Ghorbanizamani (2024)	7
Transparency and traceability	These have been major challenges in the food supply chain before the implementation of AI. Blockchain solutions can help improve data tracking and interoperability while respecting privacy, leading to increased public trust and quality control	Abid et al. (2024), Mu et al. (2024), Talari et al. (2022), Onyeaka et al. (2024), Yuvaraj et al. (2023), and Moulahoum and Ghorbanizamani (2024)	6
Collaboration and stakeholder engagements	A global approach is needed, involving FS experts, computational specialists, ICT implementation, and government interventions. Stakeholder engagement can face reluctance due to cultural differences and communication barriers. Data sharing remains a significant challenge	Onyeaka et al. (2024), Yuvaraj et al. (2023), Chen et al. (2023), Raki et al. (2024), Mu et al. (2024), and Moulahoum and Ghorbanizamani (2024)	6
Affordability and cost efficiency	Initial setups and maintenance can be resource‐intensive both economically and energetically. This can pose a great challenge for small‐scale food producers	Aggarwal et al. (2024), Inglis et al. (2024), Onyeaka et al. (2024), Talari et al. (2022), and Mu et al. (2024)	5
Big data	The 5Vs of big data—Volume, velocity, variety, value, and veracity. It also includes challenges related to data storage (cloud computing), security, and live data sources	Abid et al. (2024), Kim and Kim (2022), Talari et al. (2022), and Moulahoum and Ghorbanizamani (2024)	4
Computational and hardware constraints	Computational power refers to the processing capability of a device. Complex AI models usually require specific hardware, high computational power, and good internet connectivity	Abid et al. (2024), Aggarwal et al. (2024), Inglis et al. (2024), and Mu et al. (2024)	4
Overfitting	Occurs when the model is too specific to the training data and struggles to generalize to new, unknown data. A good model should be able to apply learned patterns to different food products, contaminants, or even locations	Abid et al. (2024), Aggarwal et al. (2024), Inglis et al. (2024), and Raki et al. (2024)	4
Model interpretability	Drawing clear conclusions can be challenging due to the black‐box nature of some deep learning algorithms. The lack of detailed hyperparameter descriptions makes it difficult to reproduce results and validate findings	Aggarwal et al. (2024), Inglis et al. (2024), and Onyeaka et al. (2024)	3
Spectroscopy data	Strong correlations between neighboring wavelengths and the complex constitution of spectral data create challenges from laboratory to practical applications. Spectroscopy techniques face issues regarding data robustness and transferability. Developing AI models that generalize well across different detection modalities and food matrices is challenging	Aggarwal et al. (2024), Raki et al. (2024), and Gul et al. (2024)	3
AI Integration with conventional methods	AI techniques must be integrated with conventional methods to ensure industrial adoption. Challenges exist in extrapolating AI applications across various industries	Aggarwal et al. (2024) and Pérez‐Santín et al. (2023)	2

*Data‐related challenges*: These were grouped into three primary sub‐categories:

‐ *Data quality and standardization*: This included issues such as inconsistent data formats and significant difficulties in data acquisition.
‐ *Big data‐related issues*: This encompassed technical barriers related to the storage and security of large‐scale datasets.
‐ *Spectroscopy specific challenges*: This referred to the high degree of correlations between wavelengths, which complicates feature selection.

*Computational and economic barriers*: Other identified limitations included the high demand for computational power and the significant economic barriers associated with implementing advanced AI infrastructure.

*Algorithmic and integration challenges*: Regarding AI models, key difficulties included overfitting, lack of interpretability, and challenges in integrating algorithms with traditional FS methods.

*Regulatory and ethical frameworks*: Furthermore, AI implementation is required to comply with the legal regulations and address ethical concerns. It was noted that these objectives can be achieved within a collaborative environment, where regulators, scientists, and other stakeholders interact to ensure a smooth and safe AI implementation. In these situations, challenges such as cultural differences and insufficient engagement must be addressed to facilitate effective cooperation.

“Data quality and standardization” was identified as the most frequently mentioned challenge across the literature. This issue was found to increase the time required for data preprocessing, a constraint that affected all data modalities, ranging from structured genomic data (NGS) to unstructured formats such as text and imagery.

Regulatory and ethical considerations emerged as the second most significant challenge. As novel techniques are developed, different ethical concerns arise, for example, the issue of using consumer purchasing data or social media data to predict FS risks, raising questions about data privacy and informed consent. To address these concerns, various legal frameworks and international entities exist. The General Data Protection Regulation (GDPR) in Europe regulates data protection and privacy issues, while the Organization for Economic Co‐Operation and Development (OECD) promotes international policies that ensure responsible AI usage, data privacy protection, and fair business practices.

Finally, it was observed that certain challenges were framed broadly, such as “Big Data,” while others were highly specific, such as “overfitting.” Overfitting is a phenomenon inherent to ML, particularly when a model is trained too closely on a limited dataset, thereby reducing its ability to generalize to new, unseen data.

## 5 Discussion

Several secondary studies were excluded from this tertiary study due to their lack of a clearly defined research methodology. Many were found to lack a systematic protocol or transparent inclusion and exclusion criteria, both of which are essential for ensuring rigor and reproducibility. Although some researchers may consider such procedures unnecessary, systematic review protocols establish minimum methodological standards. These standards strengthen the reliability and comparability of findings across studies. allowing for a more robust synthesis of the field. The absence of these protocols in the excluded literature highlighted a significant variance in the quality of evidence currently available in the domain of AI for FS.

One of the main threats to validity in this study relates to the quality assessment of secondary sources. Many candidate papers were found to lack sufficient information about their methodology, which limited the ability to apply strict quality thresholds. Consequently, a decision was made to include only reviews that attained a quality score greater than zero. To minimize subjectivity during the evaluation process, the final selection of articles was validated by a second reviewer validated the final article selection.

This tertiary study does not involve an in‐depth, individual analysis of every primary study selected by the 15 included secondary studies. However, relevant data on FS hazards and AI algorithms used were extracted to provide a comprehensive overview of the following trends in the field of FS and AI. Furthermore, a rigorous filtering process was applied to exclude primary studies that focused on topics outside the research scope, such as FQ or FF.

As a result, the number of primary studies referenced in the review methodology of each selected secondary study (Table 4) did not necessarily align with the total data analyzed in this tertiary study (RQ2.4 and RQ2.5). An example of this gap is the lack of information available on significant FS issues, such as temperature‐control events. This topic was specifically addressed by Yuvaraj et al. (2023), who discussed the usage of IoT sensors to monitor and regulate warehouse conditions to extend shelf life and ensure high‐quality fruits and vegetables. The same review also described the use of ANN to estimate the temperature of fruits and vegetables as an alternative strategy for reducing food waste. However, these examples primarily focused on FQ and food waste rather than on FS hazards, which were considered outside the defined scope of this research. Consequently, temperature‐control aspects were not incorporated into the present analysis. This distinction highlights the challenge of isolating pure FS data from the broader contexts of FQ and supply chain management.

Another potential threat to validity arose from the varying volume of primary studies across the selected secondary articles, combined with the application of topic‐based filtering. Secondary studies that included a higher number of primary articles may be more likely to mention more challenges or algorithms. Consequently, this may have influenced the representativeness of the extracted data by giving greater weight to reviews with larger sample sizes.

Fruits and vegetables, dairy, meat, and crops emerged as the most prominent topics in the intersection of FS and AI. This prevalence was likely attributed to the extensive regulatory frameworks governing these food categories, which resulted in the generation of large and comprehensive datasets. For instance, the dairy industry in the Netherlands was cited as a significant example; programs such as The Dutch Dairy Data Warehouse (DDDW) encouraged farmers to take real‐time data on milk production, environmental variables, and livestock health. Furthermore, organizations such as the European FS Authority (EFSA) mandate extensive FS data collection across member states. These datasets, when integrated with external variables such as meteorological information and fluctuations in market prices, can be processed by AI algorithms to significantly enhance FS predictive capabilities. The availability of such high‐volume, structured data was found to be a primary driver for the concentration of AI research within these specific food sectors.

As previously noted, the acquisition of sufficient and reliable data was identified as a critical factor that affects, for example, the type of products analyzed. Regarding the classification made in this study, “sensing data” consisted of structured formats of data retrieved through advanced technologies or devices. Besides being the most frequently utilized data type in the reviews, these data can sometimes be difficult to interpret. “open‐source databases” were also quite used; however, they often suffer from a lack of standardization and challenges related to the Findability, Accessibility, Interoperability, and Reusability (FAIR) data principles. Such inconsistencies were found to complicate the data preprocessing phase significantly. Finally, “text data” was the least popular one, probably due to the lack of standardization, the necessity of highly specialized NLP models, and privacy issues and legal restrictions.

Regarding the algorithms employed, a strong prevalence of supervised learning methods was observed, sometimes combined with an unsupervised technique such as PCA. Among these, NNs emerged as the most popular approach, especially in chemical FS hazard detection. Similarly, SVM and KNN were frequently employed in classification tasks, particularly in detecting microbial contamination and forecasting contamination levels.

Tree‐based models, such as DT and RF, were widely adopted. DTs were found to be more common in biological FS hazard classification due to their simplicity. Consequently, RF was preferred for chemical FS hazards, as it leverages an ensemble approach to manage higher data complexity.

Finally, transformer models were identified as the least frequently applied architecture. Although these models, which belong to the NN family and incorporate attention mechanisms, have shown significant success in NLP; their application in the FS domain remains limited. This is likely due to the scarcity of text‐based data, which was previously confirmed by the insights extracted from research question RQ2.2.

Regarding the challenges documented in the reviewed articles, “data quality” was identified as the most significant hurdle. However, a prominent limitation experienced during the execution of this tertiary study was “model interpretability.”

An essential aspect of systematic reviews in the field of AI is the inclusion of detailed information on the specific algorithms, validation metrics, and performance results of the primary studies they analyze. Providing this level of detail would significantly enhance the value of the reviews, as researchers would be able to identify connections between specific AI techniques and the type of data or the specific case they address.

Finally, this tertiary study considered the selected review articles and did not examine the primary studies referenced by them. If any errors existed in the documentation of results within the secondary studies, they will be reflected in our study as well.

### 5.1 Future Work

Looking ahead, the rapid advancement of generative AI technologies offers promising new directions for the field. Future studies will likely make greater use of textual data from organizations, regulatory agencies, social media, consumer feedback, and scientific publications. The development of domain‐specific large language models (LLMs), fine‐tuned on FS sources, could substantially enhance knowledge management and decision support in this domain. Such models may enable small and medium enterprises to remain up to date on food recalls and regulatory changes, automate internal documentation, and assist authorities and research institutions in conducting faster, more accurate searches and analyses, particularly when integrated with reliable, standardized data infrastructures.

## 6 Conclusions

This study presented the findings of a tertiary study on the application of AI within the domain of FS. The primary objective was to address the defined research questions and provide a comprehensive overview based on the analysis of 15 selected secondary studies.

The findings indicated that dairy products attained the highest level of research attention, suggesting that research intensity is driven not only by food relevance but also by the availability of measurable indicators and established monitoring practices. “Sensing data” emerged as the predominant data modality. This prevalence is attributed to the capacity of sensing data to convey both chemicals and microbiological data for contamination analysis, as well as sensor‐based metrics for assessing freshness and quality.

Furthermore, the results revealed that the majority of AI applications focus on the detection of chemical FS hazards, while biological, physical, or general predictive analysis remain comparatively underrepresented. This highlights a bias toward hazards that are more easily quantified but also identifies a substantive gap for future AI‐driven research in the field.

NNs emerged as the most widely used AI algorithms and the most mentioned challenges were data quality, data standardization and regulation compliance, this suggest that progress in this area is constrained less by algorithmic choice than by data reliability and interoperability. Accordingly, improvements in methodological standardization may yield greater benefits than incremental changes in model architecture alone.

The research methodology applied in this study was transparent and designed to be reproducible by other researchers. However, one of the main limitations encountered was the lack of systematic methodological rigor in many of the reviewed secondary studies. This required adjustments in the way quality assessment was performed. Furthermore, several reviews focused on FS and covered other topics within the agri‐food sector, such as FF, food waste, food availability, and climate change, meaning that FS‐specific insights are frequently embedded within a multitopic scope. In these instances, a specific filtering protocol was implemented, whereby only examples directly pertaining to FS were analyzed. This underscores the need for more unique FS‐specific syntheses or reviews.

## Author Contributions

**Marina Arribas Lopez**: writing – original draft, methodology, validation, visualization, writing – review and editing, data curation, investigation, conceptualization, software, formal analysis. **Yamine Bouzembrak**: conceptualization, methodology, validation, visualization, writing – review and editing, supervision, software, resources, formal analysis. **Bedir Tekinerdogan**: conceptualization, writing – review and editing, methodology, validation, visualization, project administration, supervision, resources.

## Conflicts of Interest

The authors declare no conflicts of interest

## Supporting information



Supplementary Information includes Table 7.
