# Supplementary material for: Artificial Intelligence in Food Safety: A Tertiary Study
Source: Compr Rev Food Sci Food Saf. 2026 Mar 14;25(2):e70443. doi: 10.1111/1541-4337.70443 (PMC12988573; doi:10.1111/1541-4337.70443)
Supplement: Supplementary file 1 — Supplementary Information includes Table 7. [file CRF3-25-e70443-s001.docx]

Supplementary information

Table 7: Online Databases and the selected articles where they were mentioned.

| Name | Description | Selected Reviews |
| --- | --- | --- |
| RASFF Food Alerts | FS alerts and notifications | (Abid et al. 2024), (Mu et al. 2024), (Wang et al. 2022) and (Talari et al. 2022). |
| GEMS Global Environment Monitoring System Food Monitoring | Global monitoring of environmental contaminants in food | (Abid et al. 2024), (Wang et al. 2022) and (Talari et al. 2022). |
| CompTox Chemical Database | Chemical toxicity and exposure database | (Abid et al. 2024) and (Talari et al. 2022). |
| European Food Consumption Repository | Data on food consumption patterns in Europe | (Abid et al. 2024) and (Talari et al. 2022). |
| ComBase | Predictive microbiology and FS models | (Abid et al. 2024) and (Talari et al. 2022). |
| World Bank Open Data | Global development and economic data | (Abid et al. 2024) and (Talari et al. 2022). |
| USDA National Nutrient Repository | Nutrient composition of foods | (Abid et al. 2024) and (Talari et al. 2022). |
| EU Pesticides Repository | Database on pesticide residues and regulations | (Abid et al. 2024) and (Talari et al. 2022). |
| FSANZ Food Standards Codes | FS and labeling regulations | (Abid et al. 2024) and (Talari et al. 2022). |
| ChemSpider Chemical Database | Chemical structure and property data | (Abid et al. 2024) and (Talari et al. 2022). |
| World Health Organization (WHO) | Global health statistics and guidelines | (Chen et al. 2023) and (Mu et al. 2024). |
| USFDA | U.S. Food and Drug Administration regulatory data | (Chen et al. 2023)) |
| ESFA (European Food Safety Authority) | FS risk assessments in the EU | (Chen et al. 2023) |
| FAOSTAT - Food and Agriculture Organization Corporate Statistical Database | Global food and agriculture data | (Mu et al. 2024) |
| NOAA National Oceanic and Atmospheric Administration | Climate and oceanic monitoring data | (Mu et al. 2024) |
| The Allergen Database | Information on allergenic food components | (Abid et al. 2024) |
| FDA iRisk (Monte Carlo Algorithm) | Risk assessment tool for FS | (Abid et al. 2024) |
| The Emilia Romagna Meteorological Service | Regional meteorological data | (Aggarwal et al. 2024) |
| MADiMa113 | Food and dietary data | (Chen et al. 2023) |
| Nutrition5k1 | Nutritional data repository | (Chen et al. 2023) |
| SAMR (State Administration for Market Regulation) | Market regulation and FS in China | (Chen et al. 2023) |
| EMA (Economically Motivated Adulteration Incident Database) | Database on food fraud and adulteration incidents | (Wang et al. 2022) |
| Eurostat | Statistical data for the European Union | (Wang et al. 2022) |
| FOSCOLLAB | Collaborative FS data platform | (Wang et al. 2022) |
| FPDI (Food Protection and Defense Institute) | Research and data on food defense | (Wang et al. 2022) |
| Chinese Quality Supervision System | Food quality control data from China | (Wang et al. 2022) |
| RIVM (Dutch National Institute for Public Health and the Environment) | Public health and environmental data from the Netherlands | (Wang et al. 2022) |
| KNMI (Royal Netherlands Meteorological Institute) | Meteorological data and climate trends | (Wang et al. 2022) |
| CFSA (China National Center for Food Safety Risk Assessment) | FS risk assessment in China | (Wang et al. 2022) |
| Chemidplus | Chemical information database | (Talari et al. 2022) |
| CCKP (Climate Change Knowledge Portal) | Data on climate change impacts | (Talari et al. 2022) |
